# Combining Laser-Induced Breakdown Spectroscopy (LIBS) and Visible Near-Infrared Spectroscopy (Vis-NIRS) for Soil Phosphorus Determination

**DOI:** 10.3390/s20185419

**Published:** 2020-09-21

**Authors:** Sara Sánchez-Esteva, Maria Knadel, Sergey Kucheryavskiy, Lis W. de Jonge, Gitte H. Rubæk, Cecilie Hermansen, Goswin Heckrath

**Affiliations:** 1 Department of Agroecology, Aarhus University, Blichers Allé 20, 8830 Tjele, Denmark; maria.knadel@agro.au.dk (M.K.); lis.w.de.jonge@agro.au.dk (L.W.d.J.); gitte.rubaek@agro.au.dk (G.H.R.); cecilie.hermansen@agro.au.dk (C.H.); goswin.heckrath@agro.au.dk (G.H.); 2 Department of Chemistry and Bioscience, Aalborg University, Niels Bohrs Vej 8, 6700 Esbjerg, Denmark; svk@bio.aau.dk

**Keywords:** water-extractable P, Olsen P, oxalate-extractable P, total P, PLSR, iPLS, CARS

## Abstract

Conventional wet chemical methods for the determination of soil phosphorus (P) pools, relevant for environmental and agronomic purposes, are labor-intensive. Therefore, alternative techniques are needed, and a combination of the spectroscopic techniques—in this case, laser-induced breakdown spectroscopy (LIBS)—and visible near-infrared spectroscopy (vis-NIRS) could be relevant. We aimed at exploring LIBS, vis-NIRS and their combination for soil P estimation. We analyzed 147 Danish agricultural soils with LIBS and vis-NIRS. As reference measurements, we analyzed water-extractable P (Pwater), Olsen P (Polsen), oxalate-extractable P (Pox) and total P (TP) by conventional wet chemical protocols, as proxies for respectively leachable, plant-available, adsorbed inorganic P, and TP in soil. Partial least squares regression (PLSR) models combined with interval partial least squares (iPLS) and competitive adaptive reweighted sampling (CARS) variable selection methods were tested, and the relevant wavelengths for soil P determination were identified. LIBS exhibited better results compared to vis-NIRS for all P models, except for Pwater, for which results were comparable. Model performance for both the LIBS and vis-NIRS techniques as well as the combined LIBS-vis-NIR approach was significantly improved when variable selection was applied. CARS performed better than iPLS in almost all cases. Combined LIBS and vis-NIRS models with variable selection showed the best results for all four P pools, except for Pox where the results were comparable to using the LIBS model with CARS. Merging LIBS and vis-NIRS with variable selection showed potential for improving soil P determinations, but larger and independent validation datasets should be tested in future studies.

## 1. Introduction

Phosphorus (P) is one of the most important nutrients for agricultural production worldwide [1]. Phosphorus is involved in vital plant physiological processes such as energy metabolism, membrane regulation and coding genetic information. Hence, P deficiency limits net primary production and crop yields [2]. Plants take up P exclusively via the soil solution from the soil P pool. In agricultural soils, the size of this pool depends on the lithogenic P content and the balance between plant P uptake and fertilizer or manure P inputs [3,4]. Various abiotic and biotic processes regulate dynamic transfers among different soil P pools as well as the exchange between the solid phase and the soil solution, thus determining the plant availability of P and its mobility in the environment [5]. In general, P is strongly retained in soils by sorption and precipitation reactions [6] or biological immobilization [7], although it can be transported through the soil by colloid-facilitated transport [8,9].

Ensuring that crops were amply supplied with P has historically provided a strong incentive for determining plant-available P in soils [10]. However, the large P accumulation that has accompanied agricultural intensification in many countries in the last century has also led to endemic P losses from soils to surface waters, causing eutrophication [11]. This provides a driver for quantifying the pool of potentially mobile soil P as part of effective mitigation [12]. Thus, numerous soil tests are routinely employed for quantifying the size of the agronomically or environmentally relevant soil P pools [13,14,15], while inventorying the total soil P resource is important for sustainable P management [16,17]. Characteristic of all these P determinations is that they are labor-intensive wet chemical analyses that often involve the use of harmful chemicals [18].

Sustainable soil P management calls for the development of new methodologies that enable measurement of the total content of P in soils and of the pools relevant for environmental and agronomic purposes. Among the different spectroscopic techniques, Laser-induced breakdown spectroscopy (LIBS) has the potential to become an alternative method for soil P determination since it offers more and better advantages compared to standard wet chemistry methods. It is a multi-element analysis that uses a short measurement time. Additionally, it only requires a small amount of material and minimum sample preparation with no use of chemicals [19]. Laser-induced breakdown spectroscopy is fundamentally an atomic emission spectroscopy that employs a high-energy laser pulse to ionize a sample, forming a plasma on the material surface. The focused laser pulses interact with the targeted sample originating a plasma consisting of partially ionized matter, which emit light when the atoms decay. The emitted light is spectrally resolved into elemental emission line spectra, where wavelengths can be associated with individual sample constituents and the size of the emission peaks to the content of the elements [20]. In soil, LIBS has been used successfully to determine total carbon content [21], trace metal concentrations [22,23,24] and macro- and micronutrients including P [25,26,27,28,29], through univariate and multivariate calibration methods. However, accurate determination of TP in soils by LIBS proved difficult when the technique was applied to a heterogeneous set of soil samples [28,30,31,32,33]. It is well known that sample heterogeneity is a challenge for quantitative LIBS determinations, and that is particularly true for soils because of their diverse and intricate composition [34]. Even though LIBS is an elemental analysis technique, several studies have shown its ability to estimate soil pH [35], humification degree of soil organic matter (SOM) [36], and soil texture [37,38] through relationships between the studied properties and the soil chemical composition [39]. For P, only one study tried to estimate plant-available soil P fractions by the use of multivariate calibration methods, in which not only spectral P signals but also signals from other soil elements affected the model. However, low coefficients of determination between 0.22 and 0.35 were obtained [33].

Besides LIBS, visible near-infrared reflectance (vis-NIRS) is another spectroscopic method that requires very little sample preparation being non-destructive. Numerous researchers have compared vis-NIRS with traditional wet chemical analysis, with promising results [40,41]. Vis-NIRS is based on the interaction between electromagnetic radiation and a substance, within the wavelength range of 400–2500 nm. The visible region of the electromagnetic spectrum (400–700 nm) is dominated by the molecular electron transitions, whereas the near-infrared region (700–2500 nm) is dominated by the overtones and combinations of molecular vibrations of the mid infrared spectral region. The absorbed radiation can be associated with different soil properties, providing qualitative and quantitative information of the sample [42]. The resulting absorption spectrum in the visible range is mainly associated with the color of SOM or oxides [43] and iron in minerals [44]. The resulting spectra have broad absorption features and overlapping bands, which are difficult to interpret. Thus, these need to be mathematically extracted from the spectra and correlated with soil properties by the use of multivariate calibrations [43]. Even though P is not spectrally active in the vis–NIR region, correlations between P-forms and spectrally active soil properties have been the basis for quantification of different P-forms indirectly by means of vis–NIR [45,46]. For soil P predictions, a number of studies have yielded contradicting results [47,48,49,50,51,52]. Stenberg et al. (2010) attributed the contradicting results to differences in the datasets in the P pool measured, but also to the P reference method used. The fate of P is strongly driven by specific soil characteristics, where P can be found in very different forms and associated with diverse soil constituents and minerals, and that makes its quantification by vis-NIRS highly soil-type-dependent [43].

In order to maximize the potential of LIBS and vis-NIRS as alternative analytical methods for quantitative determination of relevant soil P pools, the combination of both should be tested. Fundamentally, LIBS generates an atomic spectrum that gives information on the total elemental P content, but also about the correlation of other soil P pools with the elemental soil chemical composition. Vis-NIRS, on the other hand, generates a molecular spectrum that gives information about how P is bound to different compounds. Combining LIBS with vis-NIRS could potentially improve soil P predictions, by taking advantage of the fundamental differences between the two techniques. The correlation between the combined spectral data and the different P pools can be established through multivariate calibration models such as partial least squares regression (PLSR). LIBS and vis-NIR spectral data contain large numbers of variables (emission lines or bands associated with wavelength) with a high degree of covariance and substantial amounts of redundant information. In order to build calibration models with the most relevant information, variable selection methods are considered beneficial as an initial step in the multivariate analysis [53]. To our knowledge, only one study examined multi-sensor fusion including LIBS and vis-NIRS to predict available P by PLSR; however, the prediction accuracy based on data fusion remained as poor as for single sensors [54]. In contrast, the fusion of these sensors has shown good potential in other applications, such as the determination of trace metals in soils, by substantially improving prediction model results compared to single sensors [55,56,57].

Thus, the main aim of this study was to explore whether the combination of LIBS and vis-NIRS improves soil P determinations. For that purpose, four characteristic P pools of different ecological relevance were identified: water-extractable P, Olsen P, Oxalate-extractable P and total P. The specific objectives were: (i) to compare LIBS, vis-NIRS and their combination to determine four different P pools in 147 agricultural soils from Denmark, (ii) to test and compare partial least squares regression models with and without the two variable selection methods—interval partial least squares (iPLS) and competitive adaptive reweighted sampling (CARS)—and (iii) to identify the most relevant wavelength regions associated with soil P determination.

## 2. Materials and Methods

### 2.1. Origin of the Soil Samples

For our study, we selected 147 samples from a wide range of agricultural soils in Denmark for their variation in P and soil organic carbon content, soil texture, and geological origin. All samples had been collected as part of earlier studies. One subset (DK) comprised 68 samples from separate fields across the country [58], while three other subsets were obtained from single fields (Figure S1). The locations at Aarup (10 samples) [59] and Saeby (19 samples) [60] were characterized by large clay gradients. The soils from the field at Soervad (50 samples) varied strongly in organic carbon content [61]. All samples had been collected at 0–25 cm depth, air-dried and sieved to <2 mm prior to storage and further use.

### 2.2. Wet Chemical P Analyses

Water-extractable P analysis was carried out according to Sissingh (1971) [62], but with the modification that we extracted 1 g of soil with 50 mL of water. Olsen P [63] was measured according to Rubæk and Kristensen (2017) [64]. Total P was determined after digestion of 0.1 g ball-milled soil in a mixture of 1 mL concentrated H_2_SO_4_ and 2 mL concentrated HClO_4_ for one hour at 250 °C on a Tecator digestion unit (FOSS A/S, Hillerød, Denmark) prior to P determination. For these methods, phosphorus was analytically determined in solution by spectrophotometry using the molybdate-blue method for water samples [65] after appropriate dilutions and adjustments of pH. Oxalate-extractable P was determined by ICP-OES (Inductively Coupled Plasma Optical Emission Spectrometry) after extraction with acid ammonium oxalate in the dark [66].

### 2.3. Laser-Induced Breakdown Spectroscopy Analyses

The prototype LIBS system used to analyze the soil samples has been described by Knadel et al. (2017) [38]. Briefly, the system was composed of a Nd-YAG laser that operated at 1064 nm, a transmission grating spectrometer and a CCD detector. The pulse energy was 0.15 mJ, the pulse length was 1 ns and the repetition rate was 1 kHz, with a laser pulse area of 350 µm^2^. Prior to analysis, 2 g of soil was air-dried and <20-mm-sieved, and then compressed into pellets by the use of a manual press (38 MPa in 5 s). During the measurement, the pelletized sample was rotated in order to obtain a new and different surface for each laser shot corresponding to 30.000 separate measurement points (averaged into one) and to a measurement time of 30 s. The resulting spectral covered a range between 175 and 430 nm, had a spectral resolution of 0.125 nm and resulted in 2041 datapoints. All measurements were performed in air.

### 2.4. Visible and Near-Infrared Spectroscopy Analyses

The method described below is based on a well-established protocol in our lab. Prior to analysis, samples were air-dried and sieved (<2 mm). Then, they were scanned with the vis-NIRS spectrometer NIRS™ DS2500 (FOSS, Hillerød, Denmark). The commercially available instrument has two detectors: silicon (400–1100 nm) and lead sulfide (1100–2500 nm), and thus covered the spectral range from 400 to 2500 nm, with a sampling interval of 0.5 nm. During the measurements, 50 g of soil was placed in a 7-cm sample cup equipped with a 6-cm quartz window. Each of the resulting spectra consist of an average of seven measurements at different spots on the sample [38,67]. The measured reflectance spectra were transformed to absorbance spectra (A = [log (1/R)]) for subsequent analysis.

### 2.5. Spectra Preprocessing

LIBS, vis-NIRS and merged LIBS-vis-NIRS spectra were preprocessed using different methods. We removed the background signal of the LIBS spectra by applying a baseline correction method with asymmetric least squares smoothing [68]. Two settings were adjusted in this method: firstly, the second derivative constraint parameter that controls the amount of curvature allowed for the baseline; secondly, the asymmetry parameter that controls the asymmetry required of the fit. After baseline removal, each row of the LIBS matrix was normalized to a unit area.

Vis-NIR spectra were corrected for multiplicative scatter and additive effects by testing different pretreatment methods. Multiplicative signal correction [69] followed by the Savitzky Golay first derivative [70] and standard normal variate transformation were applied [71].

Additional processing was required for combining LIBS and vis-NIRS spectra into a single dataset since spectra from LIBS and vis-NIRS have fundamental differences. LIBS is based on light emission and vis-NIRS is based on light reflectance. Therefore, both spectral outputs were normalized to a unit area and mean-centered prior to applying PLSR and variable selection methods. We performed all the statistical analyses with the R software [72].

### 2.6. Variable Selection

Variable selection methods have the purpose of improving model performance by removing irrelevant variables from the spectra and reducing model complexity. Two methods were selected for variable selection in this study—interval PLS and CARS. The first method is widely used for variable selection in NIR spectroscopic data [73], while CARS is used in different spectroscopic applications [74].

While the iPLS method selects non-overlapping intervals of variables with the smallest prediction error in a PLSR model, the CARS method selects a subset of key variables based on the wavelengths associated with large absolute regression coefficients in a PLS model.

The iPLS method divides the spectra in different intervals, where a cross-validation PLSR model is conducted on each of them. The interval that obtains the lowest prediction error is then modelled again adding the remaining intervals, one by one, until the error no longer decreases [73]. We used a number of intervals of 200 variables for LIBS and 400 variables for vis-NIRS. The iPLS variable selection was performed for each soil P method individually.

The CARS method uses the absolute regression coefficients values of a PLSR model to evaluate the importance of each variable. The algorithm selects N subsets of wavelengths from N Monte Carlo sampling runs in an iterative way, based on the importance of each variable. Next, a fixed ratio of samples is randomly selected to establish a calibration model. Based on the regression coefficients, a double step wavelength selection procedure (consisting of adaptive reweighted sampling and the exponentially decreasing function) is used to select the most relevant variables. Ultimately, the subset with the lowest root mean square error is chosen after applying cross validation [74]. We used 500 Monte Carlo sampling runs. The CARS variable selection was performed for each soil P pool individually in Matlab software version R2019b [75].

### 2.7. Regression Modeling for P Determination

PLSR was used to correlate LIBS, vis-NIRS and combined LIBS-vis-NIRS soil spectra with the reference data of Pwater, Polsen, Pox and TP separately. We performed all the statistical analyses with the R package “mdatools” [76].

We developed calibration models using all 147 samples. All calibration models were built based on 10-fold cross-validation. The selected cross-validation method enabled us to make use of all samples and to make a direct comparison between the two spectroscopic techniques and their fusion. For the PLSR models’ evaluation, the Root Mean Square Error of Cross Validation (RMSECV), R^2^, and the bias were used. Additionally, the standardized RMSE (SRMSE = RMSE/range) was used to be able to compared the model performance between different determination methods with different P concentration ranges.

Part of the procedure for building PLS calibration models is to choose the optimal number of latent variables (LVs) or factors. Each additional LV will cause the model to explain an increasingly higher percentage of the cumulative variance in Y, the reference data. However, including too many LVs can result in overfitting. Thus, two criteria for choosing the optimal number of LVs were followed. Firstly, the number of LVs corresponding to the point at which RMSECV no longer decreased considerably was chosen [77]. Secondly, if an additional LV explained less than 2% of the variance in Y, the prior number of LVs was chosen.

Finally, depending on the model, between one to four outliers were found and discarded during the PLSR modeling process following the Rodionova and Pomerantsev (2020) method based on the total distance for every sample in projection modeling [78].

## 3. Results and Discussion

### 3.1. Soil Samples and Their Characteristics

The 147 agricultural soils samples collected across Denmark exhibited wide ranges for the different soil P pools [3]. Polsen had a larger range of 74 mg P kg^−1^ than Pwater (21.8 mg P kg^−1^), but their coefficients of variation were similar (Table 1). The Pox and TP had much larger ranges of 870 and 882 mg P kg^−1^, but lower coefficients of variation compared to Pwater and Polsen. Pwater, Polsen, Pox and TP have also been measured in 50 different agricultural soils from Austria and Germany [79]. In that study, the samples were chosen to cover a broad spectrum of soil properties and P levels. The ranges for Pwater, Polsen, Pox and TP were approximately 10, 80, 800 and 900 mg P kg^−1^, respectively, which is in line with those in this study; thus, we considered the reported P ranges (Table 1) to be representative of central and northern European agricultural soils.

Finally, the 147 samples exhibited wide ranges in texture and soil organic carbon (SOC), where soil texture varied from very sandy (approx. 3% clay) to clayey (approximately 42% clay) (Figure S2), with a high gradient in SOC ranging from 0.9% to 6.6% (Table 1, Figure S3).

### 3.2. LIBS Results

#### 3.2.1. PLS Regression Models

Prior to calibration model development, different baseline and normalization methods were tested and the best results were obtained using asymmetric least squares correction with normalization to unit area. Since the goal of this study was a method comparison and the sample set was rather small and highly variable, a cross-validation was applied.

PLSR models with full spectra yielded Rcv^2^ ranging from 0.12 to 0.67 for the different P pools with the lowest Rcv^2^ obtained for TP. The obtained errors (RMSECV) were 3.9, 9.5, 81.7 and 131.7 mg P kg^−1^ for Pwater, Polsen, Pox and TP, respectively. LIBS has previously been tested for the quantification of TP in soil as well as plant-available P. However, the plant-available P was obtained with other methods than Polsen. A recent study used PLSR to estimate TP in 200 samples based on four different soil types (paddy, Fluvo-aquic, red and black soils) and obtained a higher RMSECV (222 mg kg^−1^; 1870 mg kg^−1^ P range) than in our study [32]. The fact that we obtained a significantly lower RMSECV could be related to the very different characteristics of the paddy, fluvo-aquic, red and black soils compared to soils used in this study. Another study used PLSR for TP and plant-available P determination on 137 samples sharing the same parent material and similar textures. The authors obtained low Rv^2^ values of 0.14 and 0.22 and RMSE of 80.4 and 3.3 mg kg^−1^ for TP and plant-available P, respectively [33]. Unfortunately, they did not report their ranges in TP and plant-available P, but only the average content of TP, which was low compared to our study.

A higher accuracy was obtained when using the iPLS variable selection than with the full spectrum. For iPLS, Rcv^2^ values were 0.62, 0.73, 0.77 and 0.72 and RMSECV 3.2, 8.8, 64.6 and 73 mg kg^−1^ for Pwater, Polsen, Pox and TP, respectively. Particularly for TP, models without variable selection showed a very poor performance compared to the models with iPLS, which resulted in a six-fold higher Rcv^2^ (Table S1). Even though some LIBS studies have used iPLS variable selection coupled with PLSR and obtained accurate predictions in diverse applications like heavy metals in soil [80,81,82,83], these did not involve soil P determination.

PLSR models with CARS variable selection resulted in an Rcv^2^ ranging from 0.64 to 0.86 for the soil P pools. The RMSECV for Pwater (2.6 mg kg^−1^), Polsen (7.6 mg kg^−1^) and Pox (50.1 mg kg^−1^) were lower than the ones obtained with the iPLS variable selection. In general, PLSR models with CARS predicted Pwater, Polsen and Pox with a larger precision than PLSR models with iPLS. In contrast, iPLS resulted in better predictions of TP (Table S1). To the authors’ best knowledge, this is the first study where CARS was used together with the application of LIBS to soil. However, CARS has been applied to LIBS data in other materials, and was able to effectively screen the relevant variables and improve calibration models [84,85].

In general, fewer variable selection methods have been applied to the atomic spectrum (LIBS) than to the molecular spectrum (NIR). One of the reasons for this is that atomic spectra usually contain tens of thousands of informative variables, which would result in extensive calculations [86], however, that is not the case in this study since the LIBS data contained half the number of variables compared to NIRS. Previous LIBS studies have used different strategies when selecting input variables for PLSR modeling for TP determination. Guo (2019) included only the characteristic P lines (213.6, 547.7 and 253.5) but did not obtain satisfying results. Xu et al. (2019) compared PLSR models using the full spectra and PLSR models using only the characteristic P lines (253.1–254.5 nm) and showed that a model based on P lines only was worse than the one based on full spectra. Erler (2019) built calibration models for TP and plant available P using the full spectra, but the obtained results were not promising. The authors concluded that testing different variable selection methods and taking into account the elements indirectly related to different P pools as well could be important, which is in line with our objectives.

Among all the models, the lowest RMSECV values for Pox and TP were around 2.5–4 times lower (Table S1) than the minimum P concentrations (Table 1). However, for Polsen and Pwater, RMSECV values were higher than the minimum concentrations (Table 1 and Table S1). This indicates a challenge for the LIBS-based estimation of Pwater and Polsen.

**Figure 1:**
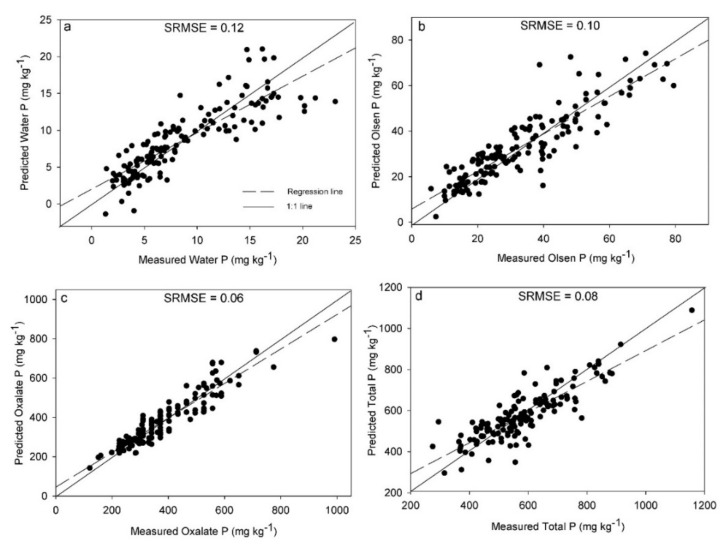
Partial least squares regression and variable selection results of the best laser-induced breakdown spectroscopy (LIBS) models, presented as the predicted versus reference measurements for water-extractable P (**a**), Olsen P (**b**), Oxalate-extractable P (**c**) and total P (**d**) measurements. Standard root mean square error of cross validation (SRMSE) is also included in each subplot.

The standard RMSEs of cross-validation (SRMSECV) can be used to compare the results obtained for each P pool. The lowest SRMSECV was obtained for Pox, followed by TP, Polsen and Pwater (Figure 1). The low SRMSECV for Pox corresponds to the highest Rcv^2^ (0.86) obtained for the prediction of this P pool. The better prediction of Pox compared to the other P pools may be related to the specific P fraction extracted. In non-calcareous soils like ours, oxalate primarily extracts P that is reversibly bound to poorly-crystalline Al- and Fe-oxides [5]. At the same time, Pox typically represents a large proportion of the TP content [79,86]. Elemental Al and Fe are characterized by high emission signal intensities in LIBS spectra [37,87,88]. Therefore, indirect correlations with the emissions of these elements and Pox concentrations in soils may have resulted in better calibration models for Pox compared to the rest of the P pools. However, the differences in model performance among the P pools could also be a result of the errors related to the different wet chemical methods for P determination used as references, and that should be considered in future studies.

#### 3.2.2. LIBS Wavelengths Selected for P Determination

**Figure 2:**
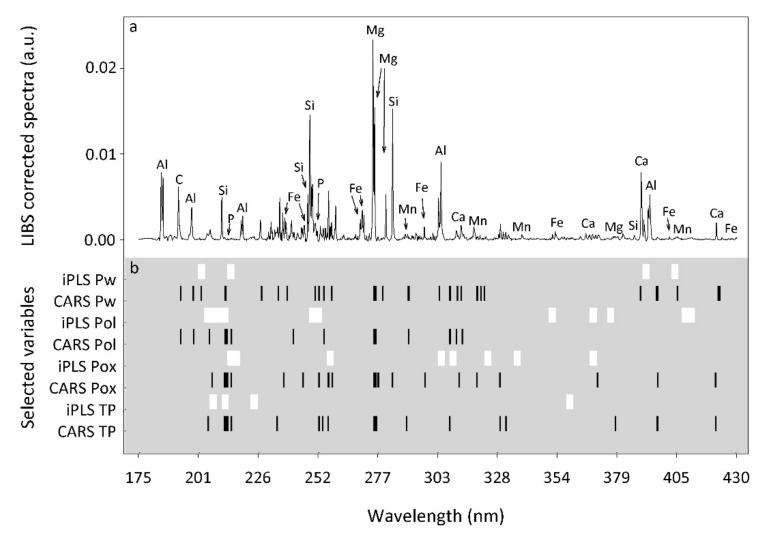
Representative LIBS-corrected spectra with marked elements associated with their emission lines (**a**) and selected variable regions (**b**) by iPLS (white) and CARS (black) for Water-extractable P (Pw), Olsen P (Pol), Oxalate-extractable P (Pox) and Total P (TP).

For iPLS, the number of LIBS selected variables ranged from 80 to 180 (Table S2). Although no single common variable was selected for all P pools, many similarities in the selected regions were observed, and wavelengths within the region 206–217 nm were selected in all cases (Figure 2b, Table S2). Earlier LIBS studies and the US National Institute of Standards and Technology (NIST) considered P emission lines at 213.6, 214.9, 215.5, 253.4, 253.6, 255.3 and 255.5 nm relevant [28,30,89,90]. This agrees well with the iPLS-selected variables between 212.09 and 217.2 nm in our study (Figure 2a, Table S2). Additionally, the commonly selected region 372.7–374.9 nm overlaps the Ca emission line at 373.7 (Figure 2a), which must be related to the Ca-P forms commonly found in agricultural soils [5]. In slightly acidic soils, as in this study, we could expect inorganic P to be associated with Ca, but also with Al and Fe [5]. However, Al and Fe emission lines were not commonly selected by iPLS for all the P pools. Examining the selected variables for the P pools separately, we found different regions within 300–400 nm (Figure 2a,b) that must be associated with Fe lines at 305.89, 358.53, 404.5, 406.36 nm and Al lines at 308.21, 309.22, 394.4, 396.75 nm [37,87,88,89].

For CARS, the number of selected variables was smaller than for iPLS and ranged from 16 to 25 among the P pools (Table S2). Similar to iPLS, a large number of common variables was found in the region 213.14–216.01 and the wavelength at 255.3 nm was also selected for three out of four P pools (Figure 2a, Table S2); all of them related to the P element [89]. Additionally, the selected variables at 279.8 and 280.4 nm might be related to the Mg emission line at 279.79 nm (Figure 2a, Table S2). Knadel et al. (2017) studied the PLSR coefficient of LIBS clay models and found the highest positive regression coefficient at 279.79 nm. The authors considered the Mg emission line as a signature for clay minerals in their soils. The clay size fraction is often important for P retention in soils. In addition to direct P sorption onto clay minerals, main P sorbents like Al- and Fe-oxides as well as P-bearing Ca minerals and organic matter are typically associated with clay minerals [6]. This could explain the importance of Mg in our calibration models.

### 3.3. Vis–NIR Results

#### 3.3.1. PLS Regression Models

For the calibration models on vis-NIR spectra, combinations of different processing methods were examined. The best results were obtained using multiplicative signal correction, followed by the Savitzky Golay first derivative and standard normal variate transformation.

Similar to LIBS, the PLSR models with full spectra showed different results depending on the P pool. Reported Rcv^2^ values for Pwater, Polsen, Pox and TP were 0.60, 0.56, 0.50 and 0.28 and RMSECV values were 3.3, 11.1, 101 and 119 mg kg^−1^, respectively (Table S3). Several studies on the application of vis-NIRS to different P pools and using full spectra were found. A study from 2007 evaluated the effect of Bray and Resins P reference methods on vis-NIRS calibrations by PLSR in 200 soil samples across Uruguay [49]. They obtained an R^2^ of 0.58 and 0.61 and RMSE of 3.78 and 2.01 mg kg^−1^, suggesting that model accuracy depends on the reference method used. In a recent study by De Souza et al. (2020) on 90 soils from the same field, PLSR models for labile, moderately labile and non-labile pools of P with reported Rv2 values of 0.42, 0.19 and 0.48, respectively, were obtained [52]. Again, the authors concluded that model accuracy depended on the P pool or method used, as we also found in our study.

Likewise, PLSR models with iPLS variable selection obtained different results for the different P pools. Pwater (Rcv2= 0.62, RMSECV = 3.3 mg kg^−1^) and Pox (Rcv2= 0.62, RMSECV = 96.2 mg kg^−1^) showed similar results to models using the full spectra (Table S3). However, significantly improved results for Polsen (Rcv2= 0.85, RMSECV = 8.3 mg kg^−1^) and TP (Rcv2= 0.55, RMSECV = 103.2 mg kg^−1^) were generated when iPLS was used compared to the models without variable selection. Studies on vis-NIRS and iPLS for soil P estimation were not found. 

PLSR models with CARS obtained Rcv^2^ values ranging from 0.54 to 0.72 for the different P pools. Additionally, the RMSECV for Pwater (2.7 mg kg^−1^), Pox (76.3 mg kg^−1^) and TP (95.3 mg kg^−1^) were reduced compared to results obtained in models with iPLS.

Overall, the best vis-NIRS models, with the lowest RMSECV and highest Rcv^2^, for Pwater, Pox and TP, were found with CARS, whereas the best model for Polsen was generated with iPLS (Table S3). A large number of vis-NIRS studies for diverse soil applications have used variable selection methods such as iPLS or CARS to select the useful spectral variables and to simplify models [91,92,93]. However, studies that compare the use of iPLS and CARS on soil properties determined by vis-NIRS have not been found. Overall, studies where variable selection strategies have been based on automated methods [45,50,51,93], which have given better results than when using the full spectra [49,52], which is in line with our findings.

**Figure 3:**
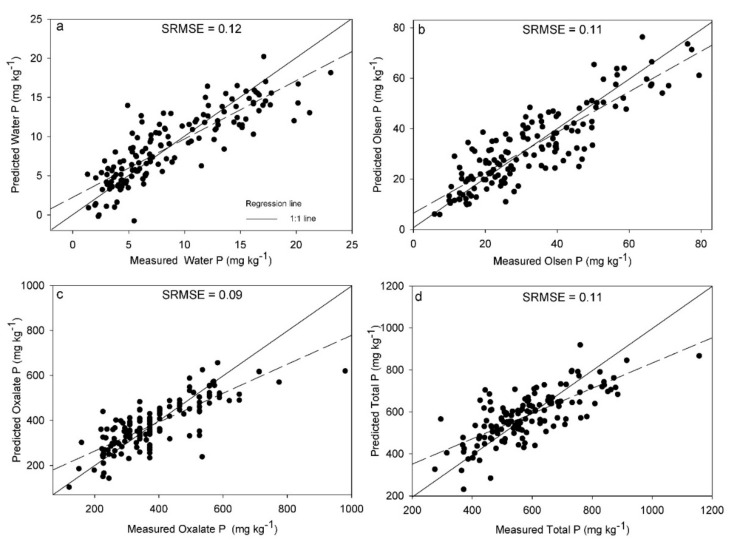
Partial least squares regression and variable selection results of the vis-NIRS models, presented as the predicted versus reference measurements for Water-extractable P (**a**), Olsen P (**b**), Oxalate-extractable P (**c**) and Total P (**d**) measurements. Standard root mean square error of cross validation (SRMSE) is also included in each subplot.

Finally, the SRMSECV produced the best results for the Pox method (0.09), followed by TP and Polsen (0.11), and Pwater (0.12) (Figure 3). Thus, similar to LIBS, Pox performed better than the other P pools. As mentioned previously, these differences can be attributed to the fraction of P measured, but also to the reference method used and its accuracy [43,49]. In general, the performance of vis-NIRS models was poorer than for LIBS with reduced SRMSECVs for all P pools, except for Pwater, which was the same (SRMSE = 0.12) for both spectroscopic techniques. Lower vis-NIRS performance compared to LIBS was expected, since the first technique is not able to sense P in soils and the calibration models are based on indirect correlations between P and other soil properties. For that reason, one could expect less robust prediction results from vis-NIRS compared to LIBS, which is in line with the results found.

#### 3.3.2. Vis-NIRS Wavelengths Selected for P Determination

**Figure 4:**
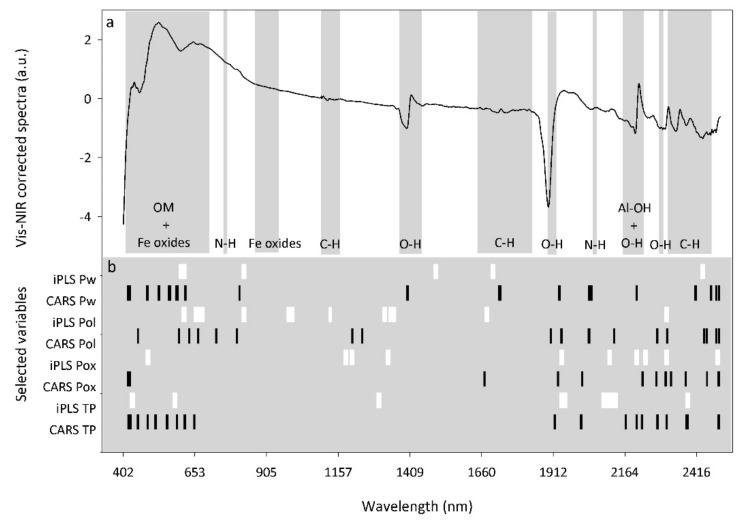
Representative vis-NIRS-corrected spectra with associated absorption bands for the different bonds present in soil (**a**) and selected variable regions (**b**) by iPLS (white) and CARS (black) for water-extractable P (Pw), Olsen P (Pol), Oxalate-extractable P (Pox) and total P (TP).

For vis-NIRS, there is no specific absorption by P; thus, differences in the reflectance shape due to P content cannot be clearly determined [45,46,48]. However, indirect correlations between P forms and spectrally active soil properties can be studied. The number of vis-NIR variables selected by iPLS for the P pools were between 116 and 315 (Table S2). Similarities between the selected variables were found mainly when P pools were separated into two groups: the most readily exchangeable P pools (Pwater and Polsen) and the more stable pools (Pox and TP) (Figure 4b). For the readily exchangeable P pools, selected regions at around 600 and 800 nm were associated with Fe-oxides and diverse functional groups of soil organic matter (Figure 4a, Table S2). The selected bands could be associated with P potentially adsorbed by hydroxyl surface groups in Fe-oxides and directly correlated with the amount of organic acids presents in soil [94]. For Pox and TP, commonly selected bands around 1900 and 2100 nm were associated with O-H and possible N-H bonds (Figure 4a, Table S2). Some of these bonds, or a mixture of them, could dominate the organic residue connected with phosphate molecules [50]. Although repeated wavebands in the visible range were not found for Pox and TP, some regions in the visible range were also relevant (423–434; 478–488; 573–585 nm) (Figure 4b).

Similar to LIBS, the number of vis-NIR variables selected by CARS was lower than for iPLS, ranging from 26 to 93 for all the P pools (Table S2). The regions between 400–600 nm and 2300–2500 nm were repeatedly selected for all the different P pools by this method (Figure 4b, Table S2). In a recent study, the most relevant variables for P models were selected based on the variable importance in the projection (VIP) method and the PLSR coefficients, similar to our study, were located around 500 and 2200–2400 nm [51]. Another study in the same year identified important Pox regions at 454–660 nm and 1732–2312 nm based on five runs of the variable selection method Generic Algorithm (GA) followed by PLSR [93]. Thus, it can be concluded that soil P tends to be associated with the visible part of the spectral range, dominated by absorption due to organic matter (OM) and electronic transitions of iron (Fe) in minerals; more specifically, the wavelengths at 454–457, 506–508, 517, 518 and 660 nm seem to be particularly relevant for Fe-oxide minerals [43,95,96,97]. Additionally, soil P tends to be associated with the higher end of the NIR spectral region where combination bands from intense fundamental vibrations of O-H, C-H bonds and Al metal-OH groups take place; specifically the wavebands at 1720, 2111 and 2300 nm are associated with organic matter (C-H bond) [44,98,99] and at around 2270 nm with gibbsite (Al-oxide mineral) [100].

### 3.4. Combined LIBS-Vis-NIRS Results

#### 3.4.1. PLS Regression Models

Phosphorus pools in soil have different correlations with elements or molecules [6]. Multivariate analyses of LIBS data should be able to determine the direct amount of P, but also other key elements related to P. Multivariate analyses of vis-NIRS data might provide complementary information on the correlation between P and the main soil properties through the molecular information. Thus, calibration models and variable selection procedures were examined on the combined spectra. Apart from the previously applied preprocessing for each technique separately, additional processing was performed to adjust values measured on different scales. Different data pretreatments were tested, including standardization, but the best performance was obtained when LIBS and vis-NIRS spectral outputs were normalized to unit area, combined, and then mean-centered.

PLSR LIBS-vis-NIRS models showed similar performances, yielding values for Rcv^2^ of 0.51, 0.53, 0.46 and 0.40, respectively, for Pwater, Polsen, Pox and TP. The reported errors (RMSECV) were, respectively, 3.7, 11.4, 104.2 and 115.8 mg kg^−1^. In comparison, the LIBS-vis-NIR model for the TP pool performed better than the models developed on full LIBS or vis-NIRS spectra, since it obtained a slightly lower RMSECV (Table S4).

The LIBS-vis-NIRS models with iPLS variable selection obtained Rcv^2^ values ranging from 0.60 to 0.85, and thus performed significantly better than the LIBS-vis-NIRS models using the full spectra. RMSECVs also decreased to 2.5, 6.7, 53.6 and 87.2 mg kg^−1^ for Pwater, Polsen, Pox and TP, respectively.

Similar results were obtained for Polsen (Rcv^2^ = 0.83, RMSE of 6.8 mg kg^−1^) and Pox (Rcv^2^ = 0.86 RMSE of 51.2 mg kg^−1^) using either the CARS or iPLS variable selection procedure. However, a significant improvement was found for the LIBS-vis-NIRS models with CARS for Pwater and TP as compared to using iPLS. For these models, Rcv2 was 0.82 for both and RMSE values were 2.2 and 58.4 mg kg^−1^, respectively (Table S4).

**Figure 5:**
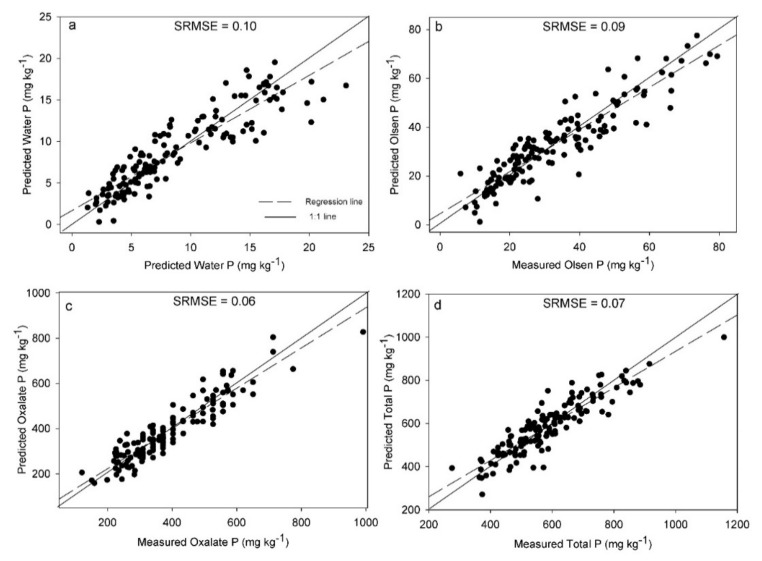
Partial least squares regression and variable selection results of the best LIBS-vis-NIRS models, presented as the predicted versus reference measurements for water-extractable P (**a**), Olsen P (**b**), Oxalate-extractable P (**c**) and total P (**d**) measurements. Standard root mean square error of cross validation (SRMSE) is also included in each subplot.

On the other hand, the lowest SRMSECV was obtained for Pox (0.06) followed by TP (0.07), Polsen (0.09) and Pwater (0.10) (Figure 5). The same trend was observed for LIBS and vis-NIRS data separately. The size of the P pool can affect the models’ performance. For example, in the Water-extractable P method, P binds to surfaces that are readily accessible to water during extraction and the P is just a minor fraction of the TP [1], which could be translated into poorer method accuracy. Additionally, the actual errors associated with the reference method used can also affect model performance and this issue should be investigated in future studies.

**Figure 6:**
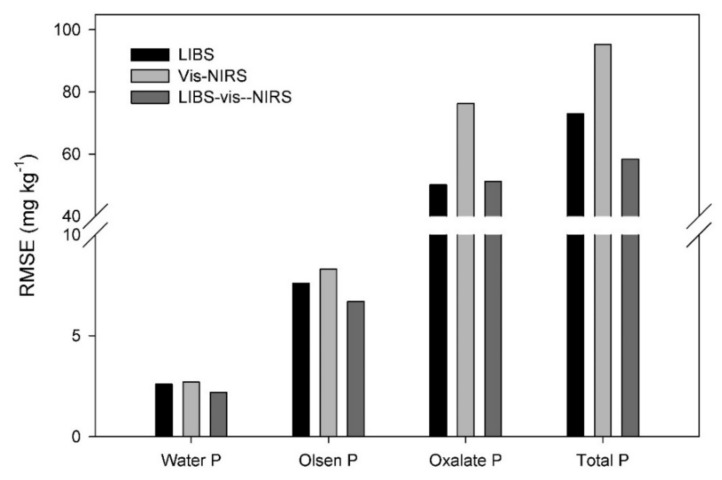
RMSE results of the best LIBS, vis-NIRS and LIBS-vis-NIRS models for water-extractable P, Olsen P, Oxalate-extractable P and total P pools. The variable selection method used is specified on top of each bar.

Overall, LIBS-vis-NIRS models using variable selection generated better results compared to LIBS and vis-NIRS separately, with the exception of Pox, for which LIBS and CARS model results were comparable (Figure 6). For Polsen and TP, the best LIBS-vis-NIRS models generated significantly lower RMSEs compared to the best LIBS or vis-NIRS models (Figure 6). We only found one study that evaluated the fusion of different sensors including LIBS and vis-NIRS, but also mid-infrared spectroscopy and X-ray fluorescence, to predict available P, among other soil properties [54]. Even though the authors found model improvements for some of the soil properties, P models obtained poor correlations and similar results as for single sensors. Another study compared vis-NIRS, LIBS, and combined LIBS-vis-NIRS for soil carbon measurements. The combined LIBS-vis-NIRS did not consistently improve soil C prediction, but the authors considered that further testing under more controlled soil conditions (intact soil core was used) and a dataset with a wider C range than the one used in the study would be necessary to determine the full potential [101].

Our study suggests that the fusion of the LIBS and vis-NIRS techniques is advantageous for the prediction of different soil P pools. When comparing the standard RMSE of cross-validation (SRMSECV) with LIBS, vis-NIRS and LIBS-vis-NIRS models, the lowest errors were obtained using the combined LIBS-vis-NIRS spectra for all the P pools (Table S4).

#### 3.4.2. Combined LIBS-Vis-NIR Wavelengths Selected for P Determination

**Figure 7:**
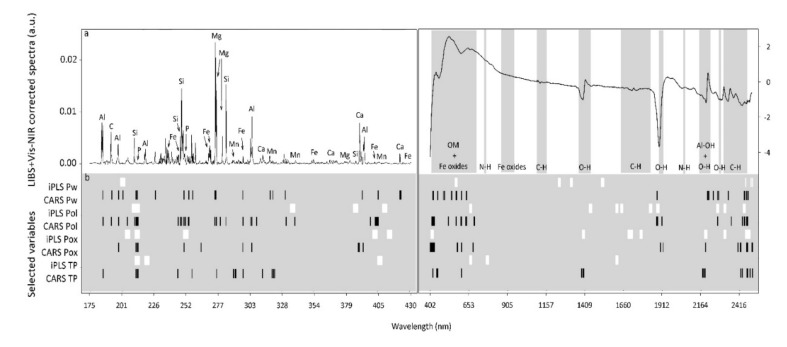
Representative LIBS-Vis-NIR-corrected spectra with elements marked above their associated emission lines and associated absorption bands for the different bonds present in soil (**a**) and selected variable regions (**b**) by iPLS (white) and CARS (black) for water-extractable P (Pw), Olsen P (Pol), Oxalate-extractable P (Pox) and total P (TP).

The number of merged LIBS-vis-NIRS selected variables by iPLS ranged from 155 to 434 for the P pools (Table S2). The most relevant selected bands appeared to be directly related to the P (I) emission lines at 213.6, 214.9 and 215.4 nm. Furthermore, the most relevant vis-NIR regions were found between 600 and 700 nm (related to Fe-oxides and diverse functional groups of soil organic matter) and around 2300 nm, which are associated with O-H bonds (Figure 7a,b).

The number of LIBS-vis-NIR selected variables by CARS were lower than with iPLS, between 80 and 132 for the different P pools (Table S2). Again, characteristic P (I) emission lines tended to be relevant, as was the wavelength at 302 nm that could be associated with the Fe (I) emission line at 301.8 nm, which is related to phosphate Fe-oxide forms commonly found in soils. In the visible range of the vis-NIRS technique, variables around 600 nm were commonly selected, indicating the potential association of phosphate with OM and/or Fe-oxides (Figure 7a,b).

In our non-calcareous soils, we would expect mineral P to be associated with Al- and Fe-oxides, which interact with OM and are mainly located in the clay size fraction [5]. Overall, most of the reported wavelengths (Table S2) were also chosen as relevant variables for the individual LIBS and vis-NIR spectra, and they were associated with characteristic P lines, organic P pools, clay minerals and Al- and Fe-oxides, as hypothesized. Therefore, we could conclude that the variable selection process for both the individual spectral methods as well as for the combined data approach was robust throughout the applied techniques and effective when screening the key wavelengths.

## 4. Conclusions

In this study, LIBS, vis-NIRS and their combination were used to estimate different pools of soil P: namely, Pwater, Polsen, Pox and TP. Additionally, the two variable selection methods iPLS and CARS were tested and compared after PLSR modeling. LIBS exhibited better results than vis-NIRS for almost all models and soil P pools. Both spectroscopic techniques and their combination showed significant model improvements when variable selection was used. The CARS variable selection method outperformed iPLS significantly, resulting in generally better model performance, and selecting, on average, five times fewer variables and showing a shorter computational time. Thus, CARS variable selection method reduced model complexity and the modeling process substantially compared to iPLS. The most relevant spectral regions for P modeling appeared to be directly related to the characteristic P (I) emission lines at 213.6, 214.9 and 215.4 nm as well as the regions within 400–600 nm, associated with Fe-oxides and diverse functional groups of soil organic matter, and at 2300–2400 nm, associated with O-H bonds.

The combined LIBS and vis-NIRS models with variable selection produced the best results for all four P pools, except for Oxalate-extractable P, where comparable results to LIBS models with CARS were obtained. Pox was also found here to have the lowest standard RMSE compared to all the other P pools. Overall, merging LIBS and vis-NIRS with variable selection showed a great potential for improving soil P determinations. To confirm these findings, larger and independent validation datasets should be tested in future studies.

## Figures and Tables

**Table 1:** Descriptive statistics of texture, organic carbon (SOC), water-extractable P, Olsen P, Oxalate-extractable P and total P for the 147 soil samples investigated in this study.

Soil Property ^1^		Mean	Max	Min	SD ^2^	CV ^3^	Skewness	Kurtosis	Q1 ^4^	Q3 ^5^
Clay	g·100g^−1^	12.8	42.2	3.3	7.1	0.55	1.81	4.29	8.2	15.2
Silt		22.5	45.9	4.5	10.5	0.47	0.02	−1.34	12.3	32.4
Sand		64.7	91.2	22.8	16.1	0.25	−0.43	−0.49	53.4	78.9
SOC		2.2	6.6	0.9	1.2	0.55	1.58	1.62	1.4	2.4
Pwater	mg·kg^−1^	8.8	23.1	1.3	5.1	0.58	0.67	−0.62	4.5	12.8
Polsen		33	79	6	18	0.53	0.73	−0.13	21	44
Pox		390	991	121	142	0.36	1.09	1.83	293	496
TP		581	1157	275	141	0.24	0.72	1.06	483	663

^1^ Summary statistics for soil texture and SOC were obtained from previous studies [58]. ^2^ Standard deviation. ^3^ Coefficient of variation (SD/mean) in%. ^4^ First inter-quartile. ^5^ Third inter-quartile.

## Supplementary Materials

The following are available online at https://www.mdpi.com/1424-8220/20/18/5419/s1, All supplemental material mentioned in the text, include Figure S1. A map containing sampling site locations in Denmark, Figure S2. The USDA soil texture triangle, Figure S3. Scatter plots between SOC and the different P fractions and Table S1. Partial least squares regression (10-fold cross-validation) results for LIBS soil phosphorus determination. Table S2. Results of variable selection methods (iPLS and CARS) presented by the commonly selected wavelengths for the P pools and their elemental assignation. Table S3. Partial least squares regression (10-fold cross-validation) results for vis-NIRS soil phosphorus determination. Table S4. Partial least squares regression (10-fold cross-validation) results for LIBS-vis-NIR soil phosphorus determination.
