# Supplementary material for: Combining Laser-Induced Breakdown Spectroscopy (LIBS) and Visible Near-Infrared Spectroscopy (Vis-NIRS) for Soil Phosphorus Determination
Source: Sensors (Basel). 2020 Sep 21;20(18):5419. doi: 10.3390/s20185419 (PMC7571271; doi:10.3390/s20185419)
Supplement: Supplementary file 1 [file sensors-20-05419-s001.pdf]

Combining Laser-Induced Breakdown Spectroscopy (LIBS) and Visible Near-Infrared Spectroscopy (Vis–NIRS) for Soil Phosphorus Determination

Sara Sánchez-Esteva [1,*], Maria Knadel [1], Sergey Kucheryavskiy [2], Lis W. de Jonge [1], Gitte H. Rubæk [1], **Cecilie Hermansen [1] and Goswin Heckrath [1]**

[1] Departmenet of Agroecology, Aarhus University, Blichers Allé 20, PO Box 50, DK-8830 Tjele, Denmark; maria.knadel@agro.au.dk (M.K.); lis.w.de.jonge@agro.au.dk (L.W.d.J.); gitte.rubaek@agro.au.dk (G.H.R.); cecilie.hermansen@agro.au.dk (C.H.); goswin.heckrath@agro.au.dk (G.H.)

[2] Department of Chemistry and Bioscience, Aalborg University, Niels Bohrs Vej 8, 6700 Esbjerg, Denmark; svk@bio.aau.dk

* Correspondence: sse@agro.au.dk

Received: 21 August 2020; Accepted: 18 September 2020; Published: date

Supplementary Material

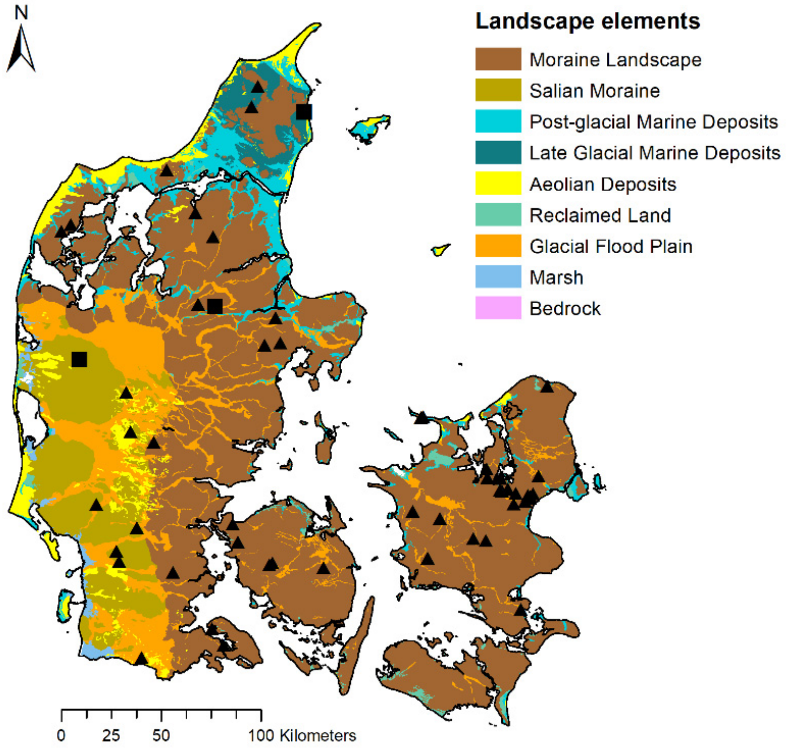

Figure S1. Location of the soil samples collected in Denmark. Triangles and squares indicate, respectively, the country-scale sample set (DK) and the sample sets from fields at Saeby (northern Jutland), Aarup (central Jutland) and Soervad (western Jutland). The underlying geological origin [S1] is shown as background.

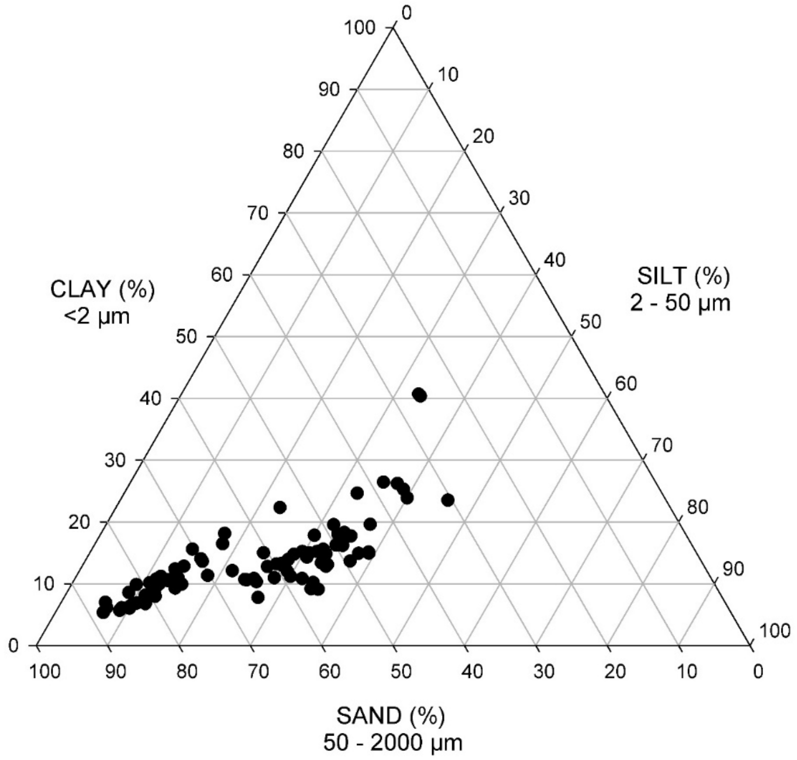

Figure S2. The USDA soil texture triangle.

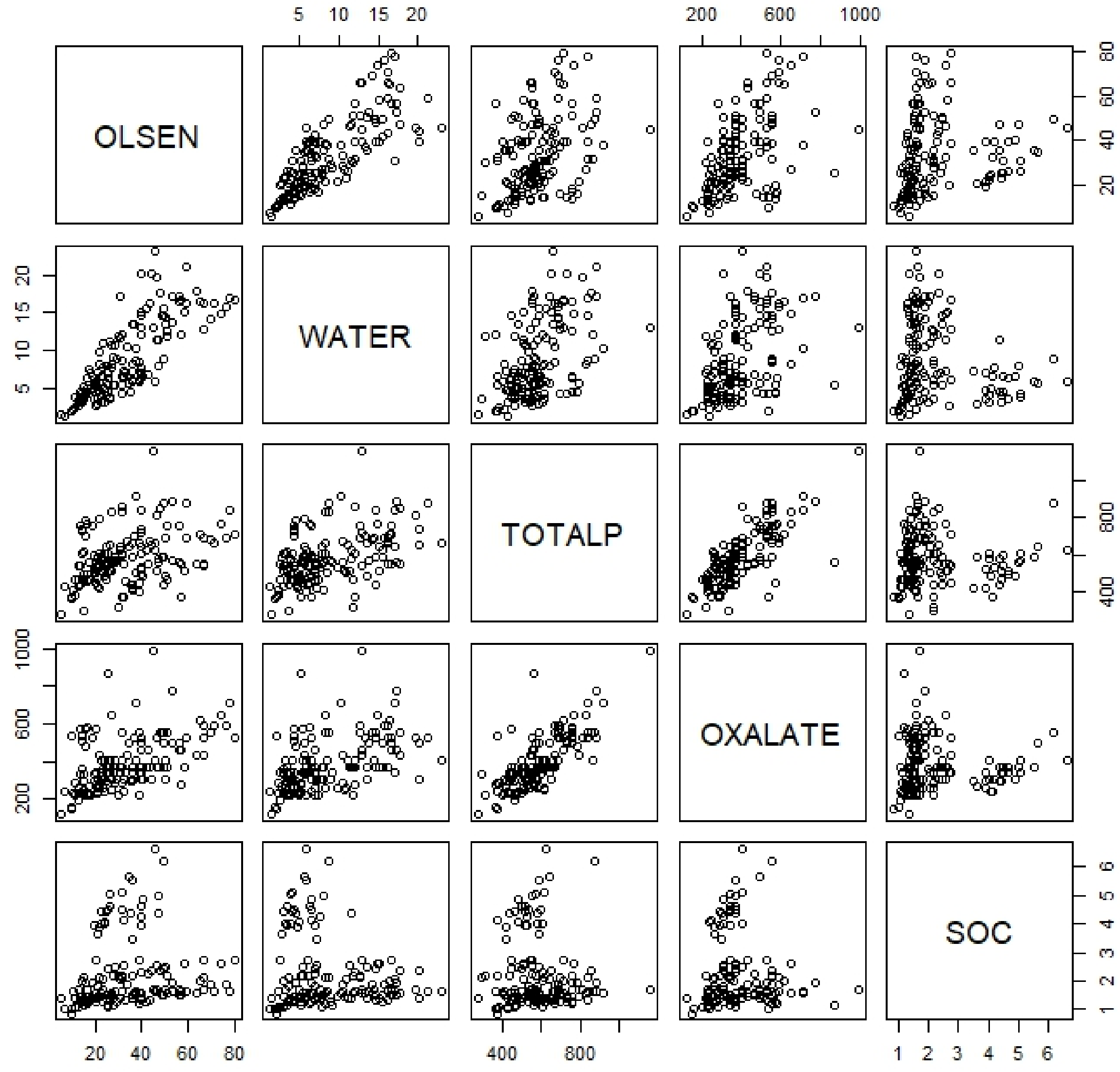

Figure S3. The linear relationship between SOC and the different P fractions; Olsen P (OLSEN) (mg kg^{-1}), Water-extractable P (WATER) (mg kg^{-1}), Total P (TOTAL) (mg kg^{-1}), Oxalate-extractable P (OXALATE) (mg kg^{-1}) and soil organic carbon (SOC) (g kg^{-1}).

Table S1. Partial least squares regression (10-fold cross-validation) results for LIBS soil phosphorus determination.

Phosphorus Ref Method	Variable Selection Method	NV[1]	RMSEC[2]	R^2	RMSECV[3]	Rcv^2	Bias[4]	NF[5]
P_{water}	None	2041	3.7	0.47	3.9	0.43	-0.01	4
	iPLS	80	3	0.66	3.2	0.62	-0.009	5
	CARS	16	2.4	0.78	2.6	0.73	0.012	8
P_{olsen}	None	2041	5.6	0.74	9.5	0.67	-0.06	10
	iPLS	180	7.9	0.78	8.8	0.73	-0.02	7
	CARS	18	6.8	0.83	7.6	0.79	0.012	7
P_{ox}	None	2041	70	0.74	81.7	0.64	-0.13	10
	iPLS	160	57.3	0.82	64.6	0.77	0.47	7
	CARS	25	44.6	0.89	50.1	0.86	1.6	8
TP	None	2041	123.2	0.23	131.7	0.12	-2.6	4
	iPLS	80	60	0.76	73	0.72	1.9	5
	CARS	25	73.6	0.73	84.8	0.64	0.95	7

[1] Number of variables (wavelengths); [2] Root mean squared error of calibration; [3] Root mean squared error of cross-validation; [4] The difference of the mean of the predicted versus the mean of the reference values; [5] Number of factors or latent variables.

Table S2. Results of variable selection methods (iPLS and CARS) presented by the commonly selected wavelengths for the P pools and their elemental assignation.

Method		Number of Selected Variables (P_{water}, P_{olsen}, P_{ox}, TP)[1]	Commonly Selected Wavelengths in nm (P pool)	Corresponding Emission Line/ Molecular Bond/Soil Component	Reference
**iPLS**	LIBS	80, 180, 160, 80	206.87 - 209.35, 212.09 - 214.58 (P_{olsen}, TP)<br>214.7 - 217.19 (P_{water}, P_{ox})<br>372.7 - 374.9 (P_{olsen}, P_{ox})	P (I)<br>Ca	Díaz et al., 2012 [28]<br>Lu et al., 2013 [30]<br>Vieira et al., 2018 [90]<br>Huang et al., 2019 [84]
**CARS**	LIBS	16, 18, 25, 25	279.78, 280.43 (all P pools)<br>213.53 (P_{olsen}, P_{water}, P_{ox})<br>214.18, 214.32, 216.01 (P_{olsen}, P_{ox}, TP)<br>213.66, 255.28 (P_{water}, P_{ox}, TP)<br>193.45, 294.97, 312.85, 313.11 (P_{olsen}, P_{water}) 213.14 (P_{ox}, TP)<br>324.76, 399.96, 400.07 (P_{water}, P_{ox})	Mg<br>P (I)<br>C	Essington et al., 2009 [S2]<br>Yu et al., 2016 [S3]<br>Díaz et al., 2012 [28]<br>Lu et al., 2013 [30]<br>Vieira et al., 2018 [90]<br>Nicolodelli et al., 2014 [S4]
**iPLS**	Vis-NIRS	116, 315, 219, 232	604.5 - 614.5, 814.5 - 824.5 (P_{water}, P_{olsen})<br>1942-1952, 2110 - 2120 (P_{ox}, TP)<br>2309.5 - 2319.5 (P_{olsen}, P_{ox})	Fe-oxides and functional groups of SOC<br>O-H and possibly N-H bonds	Stenberg et al., 2010 [43]<br>Clark et al. 1999 [44]
**CARS**	Vis-NIRS	93, 40, 26, 63	413.5 - 414, 419.5, 420.5 (P_{ox}, P_{water})<br>414.5, 584 - 585.5 (P_{water}, TP)<br>2042.5 - 2043.5 (P_{olsen}, P_{water})<br>2282,2315.5 (P_{olsen}, TP)<br>2496, 2496.5, 2497.5 (P_{ox}, TP)<br>2498 (P_{olsen}, P_{ox}, TP)	Fe-oxides and functional groups of SOC<br>O-H and possibly N-H bonds	Stenberg et al., 2010 [43]<br>Clark et al., 1999 [44]
**iPLS**	LIBS-vis–NIRS	186, 403, 434, 155	212.6 - 216.5 (P_{olsen}, P_{ox}, TP)<br>655 - 670, 1615 - 1630 (P_{olsen}, TP),<br>2312.5 - 2327.5 (P_{olsen}, P_{ox})	P (I)<br>Fe-oxides and functional groups of SOC<br>O-H bonds	Díaz et al., 2012 [28]<br>Lu et al., 2013 [30]<br>Vieira et al., 2018 [90]<br>Stenberg et al., 2010 [43]<br>Clark, 1999 [44]
**CARS**	LIBS-vis–NIRS	86, 132, 90, 80	213.5-213.8, 214.9, 302, 602.5-604 (all P pools)<br>198.9, 253.5, 416.5, 421.5-422, 2459 -2460 (P_{olsen}, P_{water} and P_{ox})<br>185.9 -186, 279.9 -280 (P_{olsen}, P_{water} and TP)<br>215.1, 2464 - 2465.5 (P_{olsen}, P_{ox}, TP)	P (I)<br>Fe (I) 301.8<br>Mg<br>Fe-oxides and functional groups of SOC<br>O-H bonds	Díaz et al., 2012 [28]<br>Lu et al., 2013 [30]<br>Vieira et al., 2018 [90]<br>Essington et al., 2009 [S2]<br>Yu et al., 2016 [S3]<br>Stenberg et al., 2010 [43]<br>Clark et al., 1999 [44]

[1] P_{water}, P_{olsen}, P_{ox}, TP refer to water-extractable P, olsen P, oxalate-extractable P and total P.

Table S3. Partial least squares regression (10-fold cross-validation) results for vis-NIRS soil phosphorus determination.

Phosphorus Ref Method	Variable Selection Method	NV[1]	RMSEC[2]	R^2	RMSECV[3]	Rcv^2	Bias[4]	NF[5]
P_{water}	None	4165	3	0.65	3.3	0.6	-0.04	5
	iPLS	116	3.1	0.62	3.3	0.6	-0.04	5
	CARS	93	2.5	0.76	2.7	0.72	-0.02	12
P_{olsen}	None	4165	10.5	0.6	11.1	0.56	-0.05	4
	iPLS	315	6.3	0.85	8.3	0.75	0.02	15
	CARS	40	7.9	0.78	9.0	0.71	-0.16	12
P_{ox}	None	4165	89	0.6	101	0.5	-0.21	7
	iPLS	210	87.2	0.62	96.2	0.54	-0.34	9
	CARS	26	70	0.7	76.3	0.64	0.2	9
TP	None	4165	110.4	0.38	119	0.28	0.61	4
	iPLS	232	94.2	0.55	103.2	0.46	0.54	9
	CARS	63	84.1	0.64	95.3	0.54	0.29	11

[1] Number of variables (wavelengths); [2] Root mean squared error of calibration; [3] Root mean squared error of cross-validation; [4] The difference of the mean of the predicted versus the mean of the reference values; [5] Number of factors or latent variables.

Table S4. Partial least squares regression (10-fold cross-validation) results for LIBS-vis-NIR soil phosphorus determination.

Phosphorus Ref Method	Variable Selection Method	NV[1]	RMSEC[2]	R^2	RMSECV[3]	Rcv[2]	Bias[4]	NF[5]
P_{water}	None	6204	3.3	0.58	3.7	0.51	-0.035	4
	iPLS	186	2.2	0.82	2.5	0.76	0.05	10
	CARS	86	1.8	0.88	2.2	0.82	0.03	11
P_{olsen}	None	6204	10.5	0.60	11.4	0.53	-0.11	6
	iPLS	403	5.8	0.88	6.7	0.84	0.0036	10
	CARS	132	5.7	0.88	6.8	0.83	0.07	11
P_{ox}	None	6204	84.5	0.64	104.2	0.46	-0.73	10
	iPLS	434	46.5	0.88	53.6	0.85	-0.74	8
	CARS	90	43.8	0.90	51.2	0.86	-2.5	9
TP	None	6204	103.5	0.45	115.8	0.40	0.60	7
	iPLS	155	70.3	0.74	87.2	0.60	-2.9	10
	CARS	80	50.5	0.86	58.4	0.82	-0.28	10

[1] Number of variables (wavelengths); [2] Root mean squared error of calibration; [3] Root mean squared error of cross-validation; [4] The difference of the mean of the predicted versus the mean of the reference values; [5] Number of factors or latent variables.

S1. Madsen, H.B.; Nørr, A.H.; Holst, K.A. *The Danish Soil Clasification. Atlas over Denmark I,3*; The Royal Danish Geographical Society: Copenhagen, Denmark, 1992.

S2. Essington, M.E.; Melnichenko, G.V.; Stewart, M.A.; Hull, R.A. Soil Metals Analysis Using Laser-Induced Breakdown Spectroscopy (LIBS). *Soil Sci. Soc. Am. J.* **2009**, doi:10.2136/sssaj2008.0267.

S3. Yu, K.-Q.; Zhao, Y.-R.; Liu, F.; He, Y. Laser-Induced Breakdown Spectroscopy Coupled with Multivariate Chemometrics for Variety Discrimination of Soil. Sci. Rep. 2016, 6, 27574, doi:10.1038/srep27574.

S4. Nicolodelli, G.; Marangoni, B.S.; Cabral, J.S.; Villas-Boas, P.R.; Senesi, G.S.; dos Santos, C.H.; Romano, R.A.; Segnini, A.; Lucas, Y.; Montes, C.R.; et al. Quantification of total carbon in soil using laser-induced breakdown spectroscopy: A method to correct interference lines. Appl. Opt. 2014, 53, 2170–2176, doi:10.1364/AO.53.002170.
